# Detection of early blight and late blight diseases on tomato leaves using hyperspectral imaging

**DOI:** 10.1038/srep16564

**Published:** 2015-11-17

**Authors:** Chuanqi Xie, Yongni Shao, Xiaoli Li, Yong He

**Affiliations:** 1 College of Biosystems Engineering and Food Science, Zhejiang University, 866 Yuhangtang Road, Hangzhou 310058, China

## Abstract

This study investigated the potential of using hyperspectral imaging for detecting different diseases on tomato leaves. One hundred and twenty healthy, one hundred and twenty early blight and seventy late blight diseased leaves were selected to obtain hyperspectral images covering spectral wavelengths from 380 to 1023 nm. An extreme learning machine (ELM) classifier model was established based on full wavelengths. Successive projections algorithm (SPA) was used to identify the most important wavelengths. Based on the five selected wavelengths (442, 508, 573, 696 and 715 nm), an ELM model was re-established. Then, eight texture features (mean, variance, homogeneity, contrast, dissimilarity, entropy, second moment and correlation) based on gray level co-occurrence matrix (GLCM) at the five effective wavelengths were extracted to establish detection models. Among the models which were established based on spectral information, all performed excellently with the overall classification accuracy ranging from 97.1% to 100% in testing sets. Among the eight texture features, dissimilarity, second moment and entropy carried most of the effective information with the classification accuracy of 71.8%, 70.9% and 69.9% in the ELM models. The results demonstrated that hyperspectral imaging has the potential as a non-invasive method to identify early blight and late blight diseases on tomato leaves.

According to a previous research, tomato fruit ranks the first place among 40 fruits and vegetables in “relative contribution to human nutrition”^1^ and is widely consumed due to its high nutrition value. Tomatoes are a rich source of lycopene, beta-carotene, folate, potassium, vitamin C, flavonoids and vitamin E^2^. They have anticarcinogenic, cardioprotective and other health benefits^3^. However, the quality and yield of tomato can be easily affected by various diseases during the growing season. Early blight (*Alternariasolani*) and late blight (*Phytophthorainfestans*) are the two common fungal diseases of tomato plants^4^. When these fungi infect tomato leaves, symptoms can rapidly spread and cover the entire leaf blade in a favorable environmental condition.

Conventionally, detecting diseases on crop leaves are based on visual assessments and diagnostic methods. Diagnose testing methods include polymerase chain reaction (PCR), enzyme linked immune sorbent assay (ELISA), fluorescence *in situ* hybridization and biomarker-based detection technology^5^. However, these methods have shown to be time-consuming, inefficient and destructive. Also, highly trained and qualified technicians are required for such detection techniques. Therefore, an advanced method is needed.

Hyperspectral imaging, which covers both spectral and imaging information, has been widely used in many fields, such as food^6^, agriculture^7^, medical science^8^, geography^9^ and archaeology^10^.The hyperspectral image (a hyperspectral cube) is composed of a series of images covering the whole wavelengths. Each pixel for one hyperspectral image has wavelengths covering the full spectral range. This technique has been studied previously for identification of diseases on crops. Williams *et al*.^11^ investigated fungal (Fusarium verticillioides) development in maize kernels using hyperspectral imaging technique. In that study, principal component analysis (PCA) was used to remove background, bad pixels and shading of the images. A partial least squares (PLS) regression model was established to predict the infection severities. Bauriegel *et al*.^12^ used hyperspectral imaging to detect Fusarium culmorum on wheat ears. PCA was used to identify four distinct wavelength ranges (500–533 nm, 560–675 nm, 682–733 nm and 927–931 nm), which allow the classification of healthy and diseased ear tissues. Spectral angle mapper (SAM) and head blight index (HBI) methods obtained correct detection rate of 84.0% and 91.0%, with an error of ±10.0%. Rumpf *et al*.^13^ detected and classified diseased sugar beet leaves based on support vector machine (SVM) classifier model and spectral vegetation indices. The eight spectral vegetation indices and soil and plant analyzer development (SPAD) were used as features for detection. Vegetation indices were related to different physiological parameters and SPAD could reflect the chlorophyll content. The detection result for infected sugar beet leaves was 97.0%. However, no texture features were studied for the above researches. Qin *et al*.^14^ discriminated citrus canker disease from healthy, insect damaged, greasy spot, melanose, scab and wind scar infected samples using the hyperspectral imaging technique. The overall detection result was 96.2% based on spectral information divergence (SID) classification method. Both spectral and imaging information were studied in this research. However, it must be noted that the SID method used in this study has limit in its applications such as on-line disease detection because of the large amount of hyperspectral data. That means optimal wavebands must be identified for on-line detection. Mahlein *et al*.^15^ diagnosed different sugar beet leaf diseases (cercospora leaf spot, powdery mildew and leaf rust) by the hyperspectral imaging technique. Spectral reflectance combined with the SAM method was used for classification. From these researches, it can be found that the hyperspectral imaging technique can be used for detecting crop disease. When the effective wavelengths were identified, a multispectral imaging detection system can be designed, which can be used in precision agriculture, therefore, on-line and non-destructive disease detection can be achieved.

This study investigated the potential of using spectral reflectance and texture features extracted from hyperspectral images to detect early blight and late blight diseases on tomato leaves. The aims of this study were: (1) to develop a technique to identify two different diseases on tomato leaves using hyperspectral imaging; (2) to select effective wavelengths for diseases identification; (3) to compare the performance of different models based on spectral and texture information; (4) to identify which wavelengths and texture features play the most prominent role for the detection of different diseases.

## Results

### Spectral analysis

#### Spectral curves

**Figure 1:**
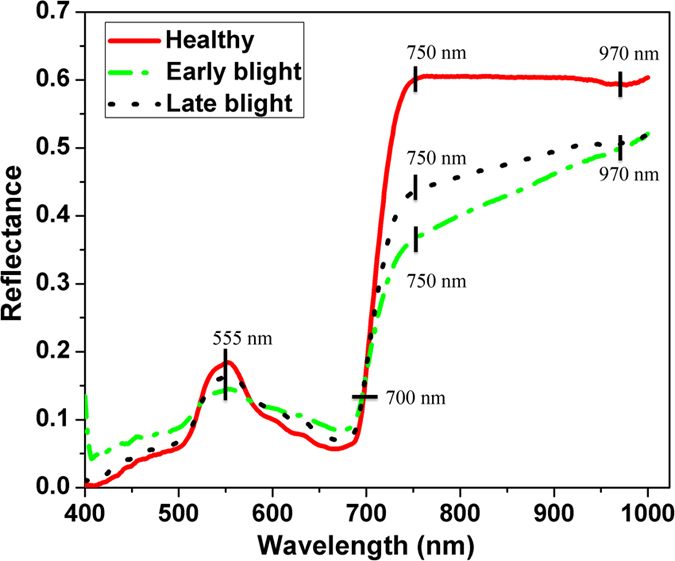
Average spectral curves of three different types of tomato leaves. For each type of leaves, the reflectance values of all samples were averaged into one value, resulting in three curves.

Mean spectral reflectance curves of healthy, early blight and late blight diseased leaves are shown in Fig. 1. The general trends of the three spectral curves were quite similar. There was a peak at around 555 nm and a valley at around 680 nm. The peak at 555 nm was the nitrogen absorption band. Reflectance increased sharply with the wavelengths from about 680 to 750 nm. Wavelength at 700 nm is the red edge. From 700 to 1023 nm, the high reflectance was due to the internal light scattering by leaf cells. The reflectance in the visible spectral region was lower than that in the near-infrared region. There were some significant differences among the samples. Reflectance of healthy samples was higher than that of infected ones in the near-infrared region (750–1000 nm), which is caused by the collapse of leaf cell structure as the disease spread. In the visible region (400–750 nm), there was some overlap between healthy and diseased leaves. Moisture content of healthy samples was higher than that of the diseased ones, resulting in obvious reflectance valley of healthy samples at 970 nm which was assigned to the O-H stretching first and second overtones^16^. There are 512 bands covering the full spectral wavelengths (380–1023 nm). However, only 400–1000 nm was studied because of the noise at the beginning and ending of the wavelengths. For wavelengths of 400–1000 nm, there are 477 bands (variables).

#### Identification results of full wavelengths

In the present study, the extreme learning machine (ELM) model was first established based on full spectral wavelengths. The spectral reflectance values were treated as *X* variables, and the sample types were treated as *Y* variables (healthy: 0, early blight: 1, late blight: 2). As shown in Table 1, the ELM models obtained satisfying results with the overall classification accuracy of 100% in both training and testing sets. However, too many input variables (477) in this method will increase the calculation time and may affect the robustness and accuracy of the model. The calculation times for full wavelengths-based and selected wavelengths-based models were 9.79 s and 1.36 s, respectively. Also, for designing a multispectral disease detection system, effective wavelengths (variables) must be selected. Therefore, the effective wavelengths were identified for establishing a simplified model.

#### Significant wavelengths

In order to simplify the model and improve the performance of the identification ability, the successive projections algorithm (SPA) was carried out to select effective wavelengths in this study. A total of five wavelengths (442, 508, 573, 696 and 715 nm) were identified as the key wavelengths by this algorithm. The selected wavelengths were then used to replace the full wavelengths for identification of different diseases. The number of selected variables suggested by SPA was only 1.05% of that of the full variables. Through wavelength selection, the raw spectral dataset was reduced to a matrix with a dimension of *m* × *x*, where *m* was the number of samples, and *x* was the number of selected wavelengths.

#### Identification results of SPA

ELM model was implemented to evaluate the detecting performance based on the effective wavelengths. The five selected wavelengths were then used to represent the full spectral wavelengths for establishing the SPA-ELM model. It performed excellently with the classification accuracy of 100% in the training set and 97.1% in the testing set. Though the classification accuracy in the testing set of the SPA-ELM model was a little lower than that of the ELM model, it is still acceptable and promising because the detection result with 97.1% is still high and it only decreased by 2.9%. Also, the calculation time decreased by 86.1% compared with ELM model simultaneously. Moreover, the number of the input variables suggested by SPA decreased largely, which has the potential to be used for developing on-line detection system. This indicated that SPA was an effective method for wavelengths selection, and the five wavelengths contained most of the effective information. The fewer input variables recommended by SPA can not only simplify the model and reduce the calculation time but also be used for designing a multispectral-based detection instrument.

#### Identification results for each selected wavelength

Each wavelength selected by SPA was used to establish identification model. There was variation in the accuracy of the ELM model depending on the wavelength used for analysis. The ELM model at different wavelengths produced different results (59.2%, 57.3%, 60.2%, 59.2% and 77.7%). The wavelength at 715 nm performed the best with the classification accuracy of 77.7% in the testing set. Other wavelengths such as 442, 573 and 696 nm also played prominent roles in the identification with accuracies of 59.2%, 60.2% and 59.2%, respectively.

### Texture features analysis

#### Texture information based on GLCM

**Figure 2:**
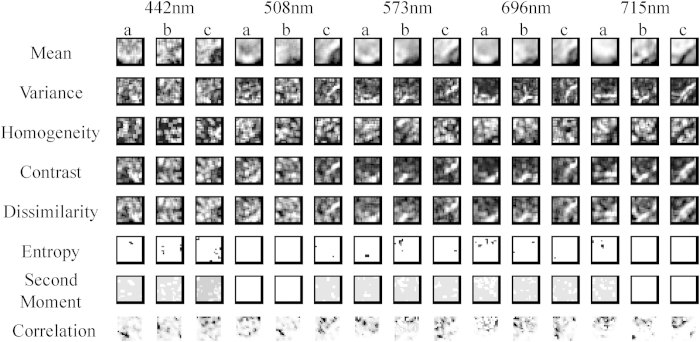
Eight texture feature images of healthy and infected tomato leaves (a: early blight; b: late blight; c: healthy). For each sample, eight texture images can be acquired at each effective wavelength. Then, texture feature was obtained from the corresponding texture image.

The hyperspectral cube (hyperspectral image) contained one image at each spectral band (a total of 512 gray images for the whole spectral bands), thus one hyperspectral image contained a large amount of redundant information which was not useful in discriminating the diseases. Therefore, images at the five selected wavelengths were used for further study. For each sample, eight texture features (mean, variance, homogeneity, contrast, dissimilarity, entropy, second moment and correlation) based on gray level co-occurrence matrix (GLCM) at five selected wavelengths (442, 508, 573, 696 and 715 nm) were extracted, resulting in a total of 40 texture features (8 texture features × 5wavelengths) for each sample. Then, identification model (ELM) was built based on these texture features. The eight texture feature images for healthy, early blight and late blight infected leaves at each wavelength can be seen in Fig. 2. It can be found different texture features had various images for the same wavelength and ROI. For the same identical texture feature and ROI, the texture images were distinct at different wavelengths. Texture images of different ROIs were also varying for the same wavelength and texture feature. This is the reason why texture features has the potential to be used for diseases detection.

#### Classification accuracies of texture features

**Figure 3:**
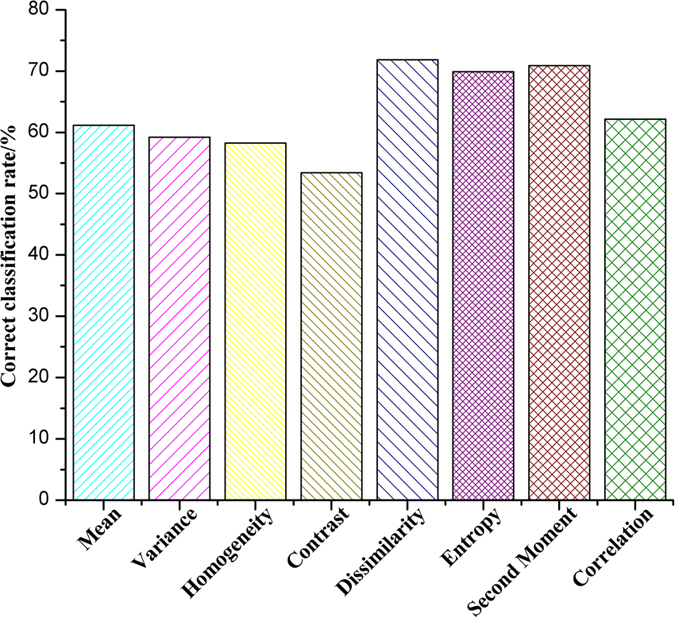
Classification accuracy of each texture feature. Each type of texture feature at the five wavelengths was put together and treated as the input variable to establish the ELM model.

The identification results based on texture features were shown in Fig. 3. Different texture features gave various results for the same model. The classification accuracies ranged from 53.4% to 71.8% in the ELM model. Among the eight texture features, the dissimilarity, second moment and entropy carried most of the effective information with high values of classification accuracy (71.8%, 70.9% and 69.9%). Also, all texture features at the five wavelengths were used for building the classification model, which obtained the classification result of 60.2%. Although results obtained using texture features are a little lower than those acquired by using reflectance, the results presented here are quite promising and encouraging for further study to detect diseases by texture features.

#### Classification accuracies for each texture feature at different wavelengths

In order to compare the performance of each texture feature at different wavelengths, the ELM models were established again. The results can be found in Table 2, which show that the same texture feature had varying classification accuracies at different wavelengths, and different texture features at the same wavelength also had varying performance. In the ELM models, the classification accuracy values ranged from 33.0% to 62.1%, 33.0% to 66.0%, 31.1% to 63.1%, 33.0% to 67.0% and 28.2% to 62.1% at the wavelengths of 442, 508, 573, 696 and 715 nm, respectively. The highest classification accuracy (67.0%) was obtained by the dissimilarity feature at 696 nm. The dissimilarity at 508 nm and 573 nm also performed well with classification accuracies of 66.0% and 63.1%, respectively. Thus, the dissimilarity feature performed the best among the eight features, and this was corresponding to the result in Fig. 3. The contrast and entropy at 508 nm also showed promising results with classification accuracies of 60.2% and 63.1%, indicating that some texture features at 508 nm performed better than them at other wavelengths. From the results, it can be found that the dissimilarity at 508 nm was the most useful texture feature for identifying early blight and blight diseases on tomato leaves. The classification accuracy for total texture feature was 60.2%, which was located in the middle of all texture features’ results. This is because some texture features such as Variance, Homogeneity, Mean and Correlation may be not useful for diseases detection, directly affected the total classification accuracy.

## Discussion

This study investigated the feasibility of using hyperspectral imaging technique to identify two different diseases on tomato leaves. Both imaging information and spectral information were investigated in this study. The ELM model was established to identify the diseased samples. SPA was applied to select useful wavelengths, which reduced the raw data. The classification accuracy was 97.1% in the testing set of the SPA-ELM model. The number of input variables suggested by SPA was five. Fewer input variables not only simplified the model but also accelerated the calculation speed. For the five selected wavelengths, reflectance at 715 nm was the most promising with the classification accuracy of 77.7%. Based on these selected wavelengths, a multispectral imaging detection system can be potentially designed, which will make the detection more efficient.

Using spectral reflectance features to detect plant diseases has been studied previously^17^,^18^. However, the spatial features were not studied because image information cannot be obtained only by using spectral technique. In our research, eight texture features (image information) extracted from hyperspectral images were studied, which has the potential to classify early blight and late blight diseases. For example, based on the gray image at the wavelength of 715 nm, the two diseases can be classified at the accuracy of 77.7%. Among the eight texture features, three (entropy, dissimilarity and second moment) performed well with high classification accuracies (70.0%, 71.8% and 70.9%, respectively). In order to study the performance of each texture feature at different wavelengths, the texture feature at each wavelength was also used to establish the ELM classification model. From the results, it can be found that the dissimilarity at 508 and 696 nm played the most prominent roles in the classification (66.0% and 67.0%). Thus, the dissimilarity may be one of the most important factor to detect early blight and late blight diseased leaves in this study. Eight texture features were extracted from hyperspectral images at the five selected wavelengths. Since there are 512 gray hyperspectral images in each sample, extracting texture features from all images is not feasible. There are two main methods to solve this problem. One is extracting texture features from several gray images at selected wavelengths, and the other is obtaining texture features from the PC images. Extracting texture features from effective images was reported by Pourreza *et al*.^19^. In that study, the authors selected the texture features from one image at the wavelength of 591 nm. However, using only one image to extract texture features may not be suitable, because some useful texture features may be in those images at other wavelengths. Also, the performance of each texture feature at different wavelengths were also not compared. The same texture feature extraction method (GLCM) was also studied by Kamruzzaman *et al*.^20^. The authors extracted four texture features (contrast, correlation, energy and homogeneity) from hyperspectral images at two wavelengths (974 and 1211 nm). However, other texture parameters such as mean, variance and dissimilarity were not studied. Extraction of texture features based on the PC images was reported by Zhu *et al*.^21^. PCA is an effective method to compress hyperspectral images. Based on the PC images, texture features can be extracted, which usually brings a satisfactory result. However, gray images those play the most important roles in the identification cannot be determined from PC images. This is because the PC images have no corresponding wavelengths, therefore, the specific gray images cannot be known.

This work demonstrated that: (1) both spectral and texture features could be used to identify the two different diseases on tomato leaves; (2) reflectance at 442, 508, 573, 696 and 715 nm were extremely important in establishing the ELM model; (3) dissimilarity at 508 and 696 nm played the most prominent roles for diseases classification; (4) entropy, dissimilarity and second moment may be the most influential texture features to identify early blight and late blight diseases on tomato leaves.

Though the results obtained by texture features were a bit worse than those obtained by reflectance, the results were quite promising and encouraging for further study. In future researches, the fusion of texture features should be used to improve the classification accuracy. Meanwhile, more samples with different infection severities should be taken into consideration for establishing more robust and accurate models.

## Materials and Methods

### Hyperspectral imaging system and software

**Figure 4:**
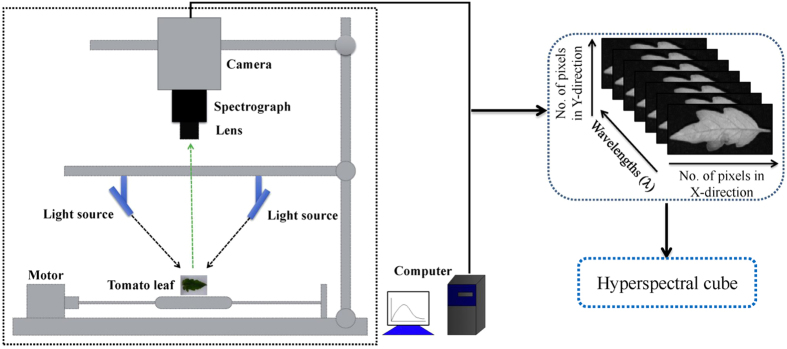
Schematic diagram of the hyperspectral imaging system (This figure was drawn by Microsoft PowerPoint). It includes a camera, a spectrograph, a lens, two light sources, a motor and a computer. This system can obtain images in the spectral region of 380–1023 nm.

A visible and near-infrared hyperspectral imaging system (Fig. 4) covering the spectral wavelengths of 380–1023 nm was used. It consists of a lens (OLE-23), an imaging spectrograph (V10E-QE, Specim, Finland), a conveyer belt operated by a stepper motor (IRCP0076, Isuzu Optics Corp., Taiwan, China), the light source from a 150 W tungsten halogen lamp (DCRIII, Schott Glass Co., Elmsford, N.Y., USA) and a computer. The area CCD array detector of the camera (C8484–05, Hamamatsu City, Japan) has 672 × 512 (spatial × spectral) pixels, and the spectral resolution is 2.8 nm. The hyperspectral imaging system scans the samples line by line, and the reflected light was dispersed by the spectrograph and captured by the area CCD array detector in spatial-spectral (*x* × λ) axes. The system itself is stationary and the sample is moving. One hyperspectral image (hypercube) can be obtained when the sample is scanned by the system. MATLAB R2009a (The Math Works, Inc., Natick, USA), ENVI 4.7 (Research System Inc., Boulder, Co., USA) and Unscrambler V9.7 (CAMO Process As, Oslo, Norway) software were used.

### Plant materials and pathogen cultivation

The variety of Zheza 809 tomato (a variety in China) was used in this study. Early blight and late blight pathogens were provided by The Institute of Biotechnology, Zhejiang University, China. The two different pathogens were cultivated on potato dextrose agar (PDA) media using different glass dishes. When the pathogen grew to about 2/3 of the glass petri dish, the hyphae on the edge were used to inoculate leaves. A small round area of about 5 mm in diameter of the hyphae was picked out using a toothpick and covered on the surface of each leaf. Each leaf was manually inoculated with a colonized agar plug. Before inoculation, all leaves were sprayed with water mist in order to make the hyphae covering the blade successfully. It must be noticed that healthy samples should also be inoculated with the agar plug without the fungi. Finally, inoculated samples with and without disease were kept in different growth chambers with the same temperature (24 °C) and humidity (90.0%) and 12 h light/dark cycle. When small disease spots appeared (about 48 hours after inoculation), a total of 120 healthy, 120 early blight and 70 late blight leaves were collected for further study.

### Flow of the study

**Figure 5:**
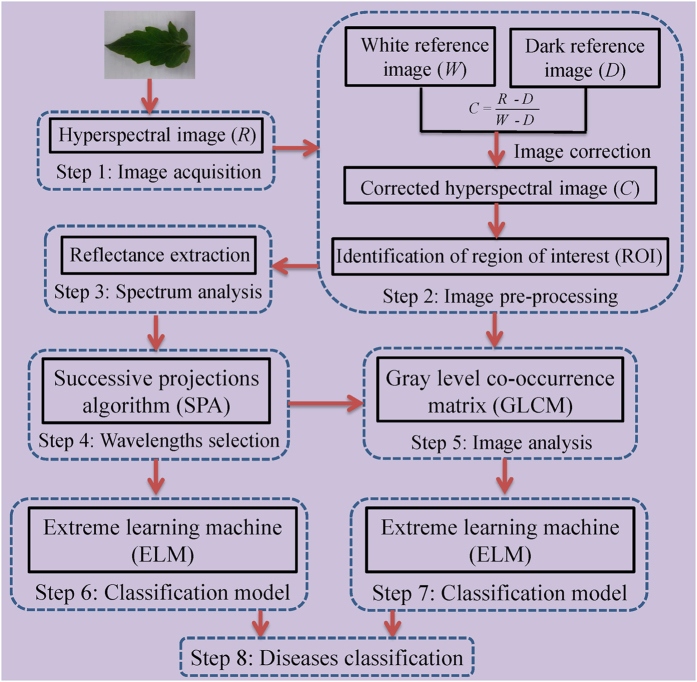
Main steps of this study. After hyperspectral images acquisition and correction, reflectance values were extracted. Successive projections algorithm (SPA) was applied to identify the effective wavelengths. Eight texture features were extracted from the gray images at the five selected wavelengths. Finally, the extreme learning machine (ELM) model was established for diseases classification.

The main steps of the study can be described in Fig. 5. Firstly, hyperspectral images of healthy and diseased tomato leaves were obtained by the hyperspectral imaging system in the spectral wavelength range of 380–1023 nm. Then, the raw hyperspectral images were corrected according to Equation (1). The reflectance values of all pixels in the region of interest (ROI) of the corrected hyperspectral images were extracted and treated as *X* variables. All samples were divided into training and testing sets at a ratio of 2:1. The ELM models were established based on full wavelengths and selected wavelengths, respectively. Eight texture features at selected wavelengths were extracted. Each texture feature was used to establish the identification model. Then, the texture feature at each selected wavelength was used to build the classification model. Finally, optimal models were identified in terms of the detection power (value of classification accuracy).

### Images acquisition and correction

Each tomato leaf was placed on the conveyer belt to be scanned line by line using the hyperspectral imaging system. The exposure time was 0.065 s while the moving speed was 3.3 mm/s. The vertical distance between the camera and the tomato leaf was 38.0 cm. A hyperspectral image was generated by using the imaging spectrograph in the spectral region of 380 to 1023 nm when the tomato leaf was scanned. The hyperspectral cube had 672 pixels in the spatial dimension and 512 bands in the spectral dimension. Raw hyperspectral images were corrected into the reference images using dark and white reference images based on the Equation (1). The dark reference image with the reflectance factor of about 0% was obtained by turning off the light and covering the camera lens with its cap. The white reflectance factor of about 99.0% was obtained from a white Teflon board (CAL-tile200, 200 mm × 25 mm × 10 mm).
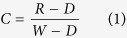
Where *C* is the corrected hyperspectral image, *R* is the raw hyperspectral image, *W* is the white reference image, *D* is the dark reference image.

### Data acquisition

#### Spectral information extraction

In order to reduce the influence of unevenness of samples and deviation of different pixels, a ROI with 20 × 20 pixels was selected manually from each leaf. For diseased leaves, the ROIs were located in the symptom areas. And the ROIs were in the corresponding areas for healthy samples. Then, the spectral reflectance values of all pixels in each ROI were averaged into one value for representing the spectral data of one sample. A total of 310 values were acquired and treated as *X* variables. Then, the total samples were divided into the training set and the testing set at a ratio of 2:1, i.e. one sample was picked out from every three consecutive samples^22^. Therefore, there were 207 samples for the training set (the numbers for healthy, early blight and late blight samples are 80, 80 and 47, respectively) and 103 for the testing set (the numbers for healthy, early blight and late blight samples are 40, 40 and 23, respectively). The spectral features for building classification models were extracted using the ENVI 4.7 software.

#### Texture features extraction

Texture, which is characterized by the relationship of the intensities of neighboring pixels, is one of the most important features in image analysis^23^. The most commonly used texture measures are derived from the grey-level co-occurrence matrix (GLCM). In this study, eight texture features such as mean, variance, homogeneity, contrast, dissimilarity, entropy, second moment and correlation were acquired based on GLCM. In the GLCM matric *P* (*i, j*), *i* and *j* are the indices of GLCM matrix elements of the five gray images. This method has been widely used in previous studies^24^,^25^,^26^,^27^. The texture information was extracted from a small area with 20 × 20 pixels which was cropped from each image. For each hyperspectral image, eight texture features were extracted at each effective wavelength suggested by SPA. The texture formulas were shown in Table 3 28. Thus, a total of 40 texture features (8 texture features × 5 wavelengths) were obtained for each sample. These texture features were treated as *X* variables to establish classification models. The texture features were calculated by the ENVI 4.7 software (Research System Inc., Boulder, Co., USA). The explanation for each texture feature can be seen as follows:
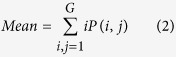
Mean stands for the average grey level of all pixels.
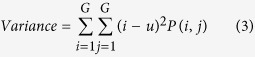
Variance indicates the rate of pixels’ value changes.
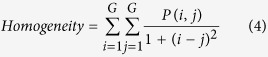
Homogeneity feature is a standard of image uniformity.
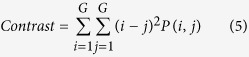
Contrast measures the local variations in the gray-level co-occurrence matrix.
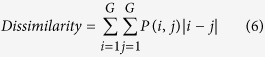
Dissimilarity stands for the difference of the grey level.
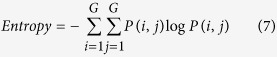
Entropy is the measurement of all the information.
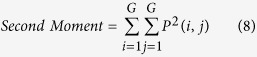
Second Moment reflects the degree of uniformity of the grey level and the size of the texture.
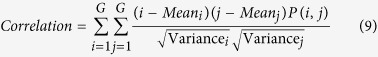
Correlation measures the joint probability occurrence of the specified pixel pairs.

#### Classification model

The ELM classification model has been used in previous studies^29^,^30^. This method, which is based on a single hidden layer neural network, has good generalization performance for feed forward neural networks. It chooses the input weights and the hidden layer biases randomly, and can solve problems such as local minima and over-fitting effectively. The learning speed of ELM was faster than that of the traditional feed forward network learning algorithm such as the back-propagation (BP)^31^. Compared with the traditional learning algorithms for neural networks, ELM tends to reach the smallest training error and norm of output^32^.

#### Effective wavelengths selection

The spectral information, covering the spectral wavelengths from 400 to 1000 nm, was characterized by high dimensionality with redundancy among contiguous wavelengths^33^. Thus, selection of significant wavelengths is very important for spectral analysis^34^. Effective wavelengths selection aims to identify a small subset of spectral features as small as possible to replace the full wavelengths. Selected wavelengths, which were conducive to design an optimized multispectral imaging inspection system, can produce results that are better than or identical to the ones received using the full wavelengths^20^.

SPA is a robust method for selecting effective wavelengths, which can resolve collinear problems by selecting optimal variables with minimal redundancy and has been used in previous studies^35^,^36^. It applies a projection operation in a vector space for the selection of effective variables with small collinearity in training^37^. For the SPA method^38^,^39^, the data are disposed in a matrix *X* = (*x*_*1*_, *x*_*2*_, *…*, *x*_*k*_) of dimensions (*N* × *K*) such that the *k* th variable *x*_*k*_ is corresponding to the *k* th column vector 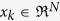. Let *M* = min (*N* − 1, *K*) be the maximum number of selected variables. In the first step, the projections are carried on the *X* matrix, which generate *k* chains of *M* variables. Each element in a chain is selected to display the least collinearity with the previous ones. The construction of each chain starts from one of the variables *x*_*k*_, (*k* = *1, …, K)*. The calculation was operated in MATLAB R2009a.

## Additional Information

**How to cite this article**: Xie, C. *et al*. Detection of early blight and late blight diseases on tomato leaves using hyperspectral imaging. *Sci. Rep.* **5**, 16564; doi: 10.1038/srep16564 (2015).

## Figures and Tables

**Table 1:** Classification accuracies of the extreme learning machine (ELM) and successive projections algorithm-extreme learning machine (SPA-ELM) models.

**Models**	**Type**	**Variables**	**Training**			**Testing**		
			**No.**^a^	**Missed** b	**CA** c**/%**	**No.**^a^	**Missed** b	**CA** c**/%**
ELM	Healthy	477	80	0	100	40	0	100
	Early blight		80	0	100	40	0	100
	Late blight		47	0	100	23	0	100
	All		207	0	100	103	0	100
SPA-ELM	Healthy	5	80	0	100	40	0	100
	Early blight		80	0	100	40	0	100
	Late blight		47	0	100	23	3	87.0
	All		207	0	100	103	3	97.1

^a^No.: number of samples.

^b^missed: incorrect identification samples.

^c^CA: classification accuracy.

**Table 2:** Classification accuracies for each texture feature at different wavelengths.

**Textural features**	**Identification of each wavelength (%)**				
	**442 nm**	**508 nm**	**573 nm**	**696 nm**	**715 nm**
Mean	48.5	56.3	54.4	58.3	54.4
Variance	33.0	33.0	31.1	33.0	28.2
Homogeneity	46.6	48.5	55.3	44.7	42.7
Contrast	53.4	60.2	58.3	57.3	54.4
Dissimilarity	58.3	66.0	63.1	67.0	61.2
Entropy	57.3	63.1	63.1	64.1	62.1
Second Moment	62.1	62.1	62.1	59.2	62.1
Correlation	48.5	49.5	31.1	55.3	59.2

**Table 3:** Expressions of texture features based on gray level co-occurrence matrix (GLCM).

**Texture features**	**Equation**	**Texture features**	**Equation**
Mean	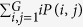	Dissimilarity	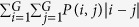
Variance	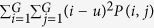	Entropy	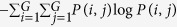
Homogeneity	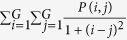	Second Moment	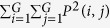
Contrast	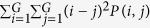	Correlation	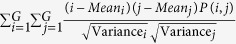

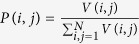
.
